# Field site-specific proteome remodeling under contrasting environmental conditions in *Brassica rapa*

**DOI:** 10.1186/s12870-026-10380-5

**Published:** 2026-11-11

**Authors:** Anastasia B. Kurina, Franziska S. Hanschen, Khabat Vahabi, Vanda Púčiková, Anna M. Artemeva, Katja Witzel

**Affiliations:** 1 N.I.Vavilov All-Russian Institute of Plant Genetic Resources (VIR), St. Petersburg, Russian Federation; 2 Leibniz Institute for Horticultural Sciences (IGZ), Theodor-Echtermeyer- Weg 1, 14979 Großbeeren, Germany; 3 Chair of Phytonutrient Management, Institute of Food Technology and Food Chemistry, Technische Universität Berlin, Kaiserin-Augusta-Allee 14, 10553 Berlin, Germany

**Keywords:** *Brassica rapa*, Environmental adaptation, Functional trait, Metabolite, Proteome

## Abstract

**Background:**

Plant plasticity enables adaptation to heterogeneous environments, yet the molecular mechanisms underlying long-term climatic acclimation in crops remain insufficiently understood. Here, we investigated the proteome plasticity of *Brassica rapa* across three contrasting field sites representing subarctic (68°N), temperate (59°N), and subtropical (44°N) climate-associated conditions. Twelve accessions spanning three crop types were cultivated, and subjected to proteome analysis and protein-metabolite correlation analysis.

**Results:**

Principal component and clustering analyses revealed a strong imprint of the field sites on proteome patterns. Functional enrichment analysis identified site-specific protein networks: subarctic conditions favored enrichment of proteins involved in photosynthesis, carbohydrate metabolism, and glycolysis, supporting energy capture and cold acclimation; temperate conditions induced proteins related to respiration and redox homeostasis; and subtropical conditions induced proteins involved in clade-specific metabolism, particularly glucosinolate biosynthesis, alongside amino acid and redox pathways. Based on proteome data, key adaptive responses to the three field sites included modulation of cellular respiration proteins, such as NADH dehydrogenase subunits, and shifts in phytohormone regulation, with cytokinins enriched under subarctic, gibberellin-regulated proteins under temperate, and auxin-associated proteins under subtropical conditions. Chinese cabbage accession A247 demonstrated the highest proteome plasticity, whereas root turnip accessions showed lower plasticity. Reaction-norm analyses across the latitudinal climate gradient revealed genotype and crop type dependent trajectories in mean protein Z-scores, which shows contrasting adaptive strategies across cultivars.

**Conclusions:**

*B. rapa* shows strong proteomic flexibility across different field sites, indicating distinct metabolic adjustments. These findings suggest that proteomic profiling aids in identifying adaptive traits and could guide crop breeding strategies to improve resilience under future climate change.

**Supplementary information:**

The online version contains supplementary material available at https://doi.org/10.1186/s12870-026-10380-5.

## Background

Adapting crops to future environments is and will remain the main challenge for crop research worldwide. High temperatures, accompanied by drought, are a major environmental factor restricting crop yields, and breeding thermotolerant crops is one key aim [1]. The application of omics techniques has led to a deep mechanistic understanding of the molecular basis of heat stress responses, however, there has been little progress in developing thermotolerant varieties [2]. One reason is the multigenic complexity of the trait. Another reason is that a vast amount of such data has resulted from greenhouse or growth chamber experiments and short-term stress treatments, and this reductionistic approach hampers the translation of knowledge into the field [3]. When compared to the growth chamber, plants grown under field conditions are continuously stressed [4] and often, a simultaneous exposure to different stress constraints occurs [5]. Even if field experiments are more challenging in terms of labor-intensity, cost, continuous fluctuations and soil heterogeneity, only the inclusion of actual field-adapted crops in omics approaches will bridge the gap between molecular mechanisms and crop performance in the field.

Plants have evolved various mechanisms to cope with abiotic and biotic stressors, and this allows the activation of physiological reactions that lead to acclimation. In order to explore the capacity of plants to adapt to changing environments, latitudinal gradients are an excellent methodological set-up as they provide information on a large temporal and geographic scale under natural conditions [6]. Numerous environmental variables differ with latitude, such as temperature, precipitation, photoperiod and light quality as well as soil characteristics and pest occurrence and due to the concurrent variations of multiple factors, disentangling single variables is challenging. Usually, latitudinal gradients have been studied in an ecological context, e.g. to understand fitness costs and other traits determining species distribution limits [7]. Here, intraspecific trait differences gave insight into adaptive genetic responses by comparing populations of the same species origin from different climates [6]. Contrasting to this, plastic responses refer to the capability of the same accessions to adjust and thrive at different climates [8], and these adjustments are reversible and not genetic. For a long time, plasticity was mainly investigated on phenotype level [9, 10], while a few studies extended plasticity to the transcriptome [11, 12]. A high level of individual plasticity is a prerequisite for surviving in unstable and fluctuating environments [13]. The range and limits of plasticity are tested in transplant experiments, covering a global latitudinal gradient. E.g., growing the same *Arabidopsis thaliana* accessions at 21 different locations across Europe demonstrated a strong latitude-dependent variation in plant secondary metabolite accumulation [14]. While such multi-location testing is undertaken in cereal research [15, 16], reports are currently limited related to vegetable research.

About 10% of the world’s vegetable production comes from the Brassicaceae, wherein *Brassica rapa* being one of the dominant species. The species encompasses a wide variety of crops including several subspecies of *B. rapa*, like leafy vegetables such as heading Chinese cabbage and non-heading pak choi and Japanese types, the turnips with tuberous hypocotyls, and broccoletto consumed for their inflorescences. A high consumption of *Brassica* vegetables is negatively correlated with developing non-communicable diseases such as cancer, cardiovascular or neurodegenerative diseases [17, 18]. Protective effects are often attributed to the degradation products of glucosinolates (GLS), a group of phytochemicals almost exclusively found in the order Brassicales, as well as other plant secondary metabolites such as carotenoids and polyphenols [19, 20]. Crop improvement relies on plant genetic diversity. The available genetic biodiversity of *Brassica* vegetables is an outstanding foundation for breeding efforts to develop climate-resilient crops [21, 22]. Numerous studies elucidated *B. rapa* germplasm diversity with respect to pest resistance [23], agronomic traits [24], or metabolite accumulation [25], among others. Since biodiversity is currently being lost at increasing rates [26], conservation and utilization of agrobiodiversity has the potential to create a variety of traits that support future food-related challenges. To strengthen the link between conservation and use of germplasm collections, a set of 100 *B. rapa* accessions was grown in 2021 at three different site-associated climatic conditions (subarctic, temperate, subtropical) and the study identified accessions with high resistance against insect pests [27]. In parallel, a core collection of twelve *B. rapa* accessions, covering three subspecies, was grown under the same conditions to investigate the range of proteome plasticity along a latitudinal gradient and these results are presented here. This core collection included accessions of three cultivar types to present maximum of species diversity: Chinese cabbage was presented by leafy cultivar type Dunganskaya (A672, from Korea), semi-heading cultivar types Santo originated from Russia and Japan (A36, A270) and Kasin from Korea (A247), heading cultivar types CheeFoo (A401, clubroot resistant from Japan), Matsushima (Avr1557 from Japan with dwarfism), cylindrical type Granat (A449 from the Netherlands); pakchoi was presented by cultivar type Yutsai (A195, from China) and Taisai (A214, from Japan); root turnip was presented by cultivar type Gelria (A1300, from Germany), Mammoth (A1409, from Russia) and Golden Ball (A1424, from Finland). All accessions are resistant to early bolting, so have been able to form the productive organ with good quality in all geographic and climatic conditions, including subarctic region. Further, accessions show different level of resistance to insects.

The objective of our study was to identify global physiological adaptations and to examine, which proteins contribute to site-specific acclimation under field conditions. Our study provides molecular background to make a step towards both predicting the effect of climate change on crops and intervening to improve those outcomes, especially with regard to production system resilience and crop yield.

## Methods

### Plant cultivation

The material for the study included twelve *B. rapa* accessions, belonging to three subspecies: Chinese cabbage (7 acc.), pakchoi (2 acc.) and root turnip (3 acc.). In the following, the genebank catalog number, crop type, accession name, cultivar type, origin and year of entry into genebank collection is provided for all accessions: (1) VIR 270, Chinese cabbage, Harumaki Shin Santousai, semi-heading, var. *infarcta* Li, Japan, 1976; (2) VIR 449, Chinese cabbage, Hoyo F1, heading, var. *cephalata f. cylindrica* Li, the Netherlands, 1989: (3) VIR 672, Chinese cabbage, IT 104056, leafy type, var. *dissoluta* Li, South Korea, 1999; (4) VIR 401, Chinese cabbage, Kanki F1 CR 106, heading, var. *cephalata f. ovata* Li, Japan, 1991; (5) VIR 247, Chinese cabbage, Khasinbechu, heading with open top, var. *laxa* Li, North Korea, 1982; (6) VIR 36, Chinese cabbage, Khibinskaya, semi-heading, var. *infarcta* Li, Russia, 1945; (7) VIR Avr. 1557, Chinese cabbage, Mini Raioh, heading, var. *cephalata f. ovata* Li, Japan, 2017; (8) VIR 195, pakchoi, Mestniy, Yutsai type, China, 1966; (9) VIR 214, pakchoi, Nikanme Jikijiro Taisai, Taisai type, Japan, 1976; (10) VIR 1409, root turnip, Afiko, mammoth type, Russia, 2019; 11) VIR 1424, root turnip, Golden Ball, mammoth type, Finland, 2021; 12) VIR 1300, root turnip, Siloga, gelary type, Germany, 1977. Additional information about the selected accessions related to known resistance traits (biotic, abiotic resistance) is provided in Supplemental Table 1.

**Fig. 1:**
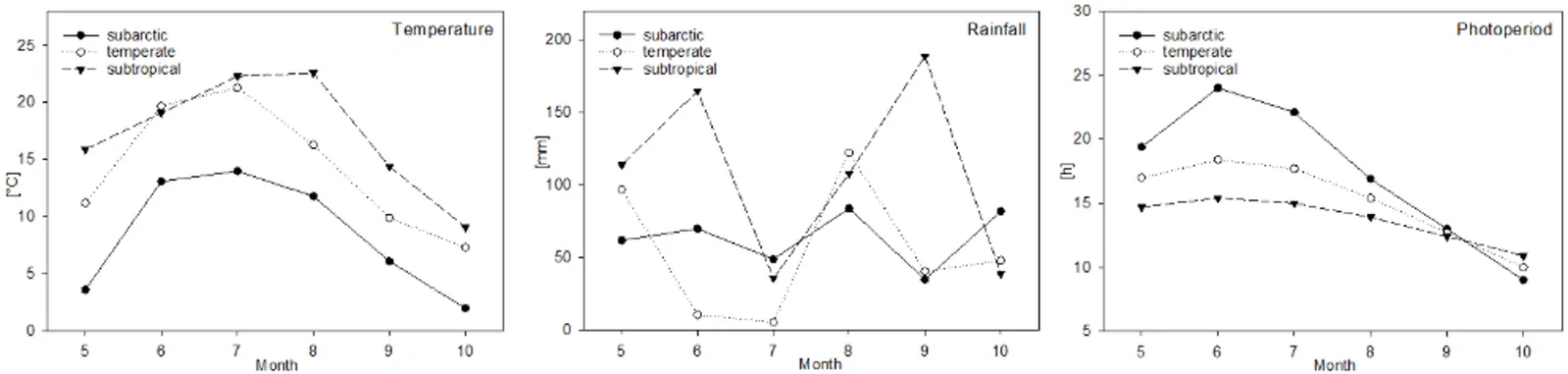
Abiotic environmental conditions of the field experimental stations in 2021 during the cultivation period. Temperature data is averaged for day and night. Sowing dates ranged from 28.05.2021 (subtropical field site) to 03.06.2021 (subarctic field site) and 09.06.2021 (temperate field site). Harvesting started at the subtropical field site on 26.07.2021, then 01.09.2021 at the temperate field site and 06.09.2021 at the subarctic field site

Sowing was carried out in three repetitions of 30 plants each at the VIR Polar Experimental Station (Far North, Murmansk region, 68°58′ N, 33°5′ E), in the following referred to ‘subarctic field condition’, Pushkin Laboratories of VIR (North-West, St. Petersburg, 59°43′ N, 30°25′ E), in the following referred to ‘temperate field condition’, and VIR Maykop Experimental Station (Southern zone, 44°36′ N, 40°5′ E), in the following referred to ‘subtropical field condition’, in the growing season 2021 [27]. Daylength, amount of rainfall and temperature during the growing period at the three experimental sites were recorded and are summarized in Fig. 1. The soil at the VIR Polar Experimental Station is characterized by podzolic, sandy, and sandy loam soils, largely boulder-laden, as well as boggy soils with poorly decomposed peat, pH was 5.0. The soil at the Pushkin Laboratories of VIR is soddy-slightly podzolic, sandy loam and loamy, with a pH of 7.1–7.6. The humus content was 2.1-3.0%, potassium was 14.3 mg/100 g of soil, and phosphorus was 72.5 mg/100 g of soil. The soil at the VIR Maykop Experimental Station is meadow-chernozem-like and meadow-forest, with heavy metal contamination from natural geological sources leading to elevated lead and cadmium content, including clays. Humus content ranged from 2.7% to 4.3%, pH was 8.0-8.3, potassium was 33.3 mg/100 g, and phosphorus was 3.5 mg/100 g of soil.

Plants were watered and fertilized according to conventional practice. Sowing dates were: 03.06.2021 (subarctic field site), 09.06.2021 (temperate field site), 28.05.2021 (subtropical field site). Harvesting dates were: 06.09.2021 (subarctic field site), 01.09.2021 (temperate field site), 26.07.2021 (subtropical field site). Plants were watered according to horticultural practice, especially at the subtropical field station. Plants for proteomic analysis were harvested in all regions, having the same developmental stage throughout the experimental stations. Plants were collected in the first half of the day from 9 to 10 am. Pakchoi and Chinese cabbage accessions were harvested when the BBCH 48 (80% of the expected head size reached) to 49 (Typical size, form and firmness of heads reached) were reached, turnips were harvested also at BBCH 48 (80% of the expected diameter of turnip reached) and 49 (expansion complete, typical form and size of turnip reached).

Feeding of herbivores *Plutella xylostella* L. (Yponomeutidae) and *Mamestra brassicae* L. (Noctuidae) was recorded using a scale to assess pest resistance/susceptibility in plants: no leaf damage or up to 5% (score 0), 6 to 25% (score 1), 26 to 50% (score 2), 51 to 75% (score 3), 76% to 100% (score 4). An average damage score for each accession was calculated as the mean of all screened plants in the accession.

### Protein extraction from plant samples and proteome analysis

Leaves of five plants were pooled to give one sample and three samples were generated in total per accession and field station. Leaf samples were freeze-dried, homogenized for protein extraction, which was performed as described earlier [28]. In short, proteins were extracted from 10 mg using RapiGest SF (Waters, Germany). Ten µg of protein were reduced, alkylated and digested with trypsin (Promega, United States) over night at 37 °C. Desalting of peptides was carried out using peptide desalting spin columns (Pierce, Thermo Scientific, United States) following the manufacturer’s instructions. Peptide concentration was adjusted to 100 ng/µL and 6 µL of protein digest were analyzed using nanoflow liquid chromatography on a Dionex UltiMate 3000 system (Thermo Scientific) coupled to a QExactive Plus mass spectrometer (Thermo Scientific). No technical replicates were performed. Peptides were separated using an Acclaim PepMap 100 C18 analytical column (75 μm × 50 cm, 2 μm, 100 Å, Thermo Scientific) and eluted via a 200 min gradient with a flow of 300 nL/min from 2% to 44% solvent B (80% acetonitrile in water with 0.1% formic acid), with solvent A being 0.1% formic acid in water, and the column temperature set at 40 °C. For electrospray ionization of peptides, a Nanospray Flex ion source was used, with spray voltage set at 1.80 kV, capillary temperature at 275 °C, and S-lens RF level at 60. Mass spectra were acquired in positive ion and data-dependent mode. Full-scan spectra (375 to 1,500 m/z) were acquired at 140,000 resolution and MS/MS scans (200 to 2,000 m/z) were conducted at 17,500 resolution. Maximum ion injection time was 50 ms for both scan types. The 20 most intense MS ions were selected for collision-induced dissociation fragmentation. Singly charged ions and unassigned charge states were rejected; dynamic exclusion duration was set to 45 s.

The raw files were processed using Proteome Discoverer 2.4 and Sequest HT engine (Thermo Scientific), searching the NCBI *B. rapa* ‘Chiifu-401-42’ reference genome sequence (released in December 2020, https://www.ncbi.nlm.nih.gov/datasets/genome/GCF_000309985.2/). Precursor ion mass tolerance was set to 10 ppm and fragment ion mass tolerance was set to 0.02 Da. To address missingness patterns, raw intensity distributions were calculated for proteome (Supplemental Fig. 1). Sensitivity analyses comparing raw profiles against left-censored median-imputed proxies were conducted to ensure that fold changes, clustering, and site-specific identifications were not artificially inflated (Supplemental Fig. 1). Further parameters for database search were: peptide tolerance: ten ppm; fragment ion tolerance: 0.02 Da; tryptic cleavage with max. two missed cleavages; carbamidomethylation of cysteine as a fixed modification and oxidation of methionine as a variable modification; minimum peptide length: 6. The result lists were filtered for high confident peptides, and their signals were mapped across all LC-MS experiments and normalized to the total peptide amount. Protein abundance quantification was carried out using the Top N average method (*n* = 3) on proteins that were detected in at least 50% of runs in the replicate group. Differential abundance was assessed using a factorial linear model incorporating Climate, Genotype, Crop-type, and the Climate x Genotype interaction. To address the unbalanced group sizes among crop types, Type-III contrasts and Benjamini-Hochberg FDR corrections were applied (Supplemental Fig. 2).

### Analysis of glucosinolate hydrolysis products

GLS hydrolysis products were analyzed as described earlier [29], with some modifications: Leaves of five plants were pooled to give one sample and three samples were generated in total per accession and field station (same sample as proteome analysis). Briefly, 25 mg of lyophilized plant material weighed into a solvent resistant vessel were mixed with 250 µL of water and incubated at room temperature for 1 h. Then, hydrolysis products were extracted with methylene chloride in presence of the internal standard benzonitrile (0.2 µmol) and analyzed by GC-MS (7890 A GC with 5975 C Inert XL MSD, Agilent Technologies Deutschland GmbH, Waldbronn, Germany) as described previously, except that both an HP-5MS Ultra Inert 30 m column (30 m × 0.25 mm × 0.25 μm) (Agilent Technologies Deutschland GmbH, Waldbronn, Germany) and a BPX5 (SGE) column (Trajan Scientific and Medical, Victoria, Australia) with the same column dimensions were used during the project. No technical replicates were performed. Compound identification and quantification were performed using Agilent MassHunter Quantitative Analysis software (Version 10.2). Data from Agilent MSD ChemStation were converted to MassHunter format using the MassHunter GC/MS Translator B.0705 2479.

### Glucosinolate analysis in pakchoi grown under different irrigation regimes

To investigate the potential effect of irrigation on GLS levels in *Brassica rapa*, an experiment was conducted using pakchoi (*Brasica rapa* spp. *chinensis* cv. Black behi) grown under controlled conditions in walk-in phytochambers (ENGIE Deutschland GmbH, Köln, Germany) at the Leibniz Institute for Horticultural Sciences (Grossbeeren, Germany). Pakchoi seeds were sown in pots (0.66 L, Ø 12 cm) filled with potting soil (Pikiererde, Einheitserde Werkverband e.V., Sinntal-Altengronau, Germany), germinated and grew at moderate conditions. Temperature ranged between 18 and 22 °C, the photoperiod was set to 12 h with max. light intensity of 490 µmol m^− 2^ s^− 1^ and daily light integral (DLI) of 12.4 mol m^− 2^ d^− 1^. Plants were irrigated equally with tap water throughout the growing period.

After four weeks, the pots were thoroughly irrigated, submerged in standing water for 1 h, and subsequently allowed to drain for 1 h to achieve the maximum water-holding capacity of the substrate. The pots were then weighed and reweighed after 24 h. Water consumption over the 24 h period was calculated for each pot, and a water supply gradient corresponding to 100, 80, 60, and 40% of the 24 h water consumption was established. In addition, a waterlogged (WL) treatment was included. The pots were subsequently irrigated daily according to the defined watering regime for seven days. The experiment was conducted in two independent experimental replicates. At each harvest, three biological replicates were sampled per treatment. Whole plants were harvested by cutting them at the soil surface and subsequently divided into halves. One half was immediately shock frozen in liquid nitrogen, freeze-dried, ground to a fine powder (particle size 0.2 mm) and stored at -20 °C until the GLS analysis by UHPLC-DAD-ToF-MS. Desulfo-GLS were extracted following the previously reported protocol [30]. Analysis of desulfo-GLS was performed by HPLC-DAD-ToF-MS (1290 Infinity II LC System with 6230 TOF LC/MS, Agilent Technologies Deutschland GmbH, Waldbronn, Germany), identification and quantification of desulfo-GLS was carried out as previously reported [30].

### Data analysis and visualization

Protein abundance data was processed with Python’s pandas library. Missing values imputed using a Minimum Determinant (MinDet) approach, with PLS-DA models validated via 7-fold cross-validation (R², Q²) and 100-iteration permutation tests. For the heatmap analysis, the data were standardized to z-scores. For K-means clustering, kmeans function in R along with the optimal number of clusters (k = 10) used and its visualizations as heatmap representation, was created using the pheatmap package in R to display clusters over expression patterns (R version 2024.9.1.394, R Studio version 4.4.3; R Foundation for Statistical Computing, 2020). For PCA, protein data was transposed so that samples were in rows and standardized using the StandardScaler in python. PCA was done with the PCA class from sklearn.decomposition, extracting the first two principal components for visualization. PLS-DA was performed using PLSRegression from sklearn.cross_decomposition subsequent of data standardization. To prevent overfitting and validate site separation, the model was evaluated using 5-fold cross-validation (Q2) and a 40-iteration random permutation test of climate labels. For K-means clustering, cluster stability and optimal cluster number selection were assessed using silhouette analysis. To avoid circular interpretation when assigning clusters to climates, functional enrichment was performed on MapMan BINNAME categories using direct, FDR-controlled statistical contrasts (Benjamini-Hochberg q < 0.05) independent of the cluster assignments.

Using Python’s numpy and pandas libraries, accession-specific plasticity was measured by calculating average of the maximum and minimum abundance of protein values across all of the climate conditions. Functional enrichment analysis was done by calculating enrichment metrics based on the ratio of expressed to background proteins. Statistical tests, such as hypergeometric tests and corrections for multiple testing of p-values, were performed using scipy and statsmodels libraries to find significantly enriched biological processes related to both protein and metabolite abundance. Multi-omics integration was performed on matched samples using joint PCA and climate-specific relevance networks (|r| > 0.5, *p* < 0.05). Pairwise protein–metabolite correlations were evaluated using Benjamini-Hochberg FDR correction (q < 0.05). To evaluate whether key protein changes align with downstream metabolites​, targeted Pearson (r) and Spearman (rho) correlations were calculated across matched samples for signature proteins (TGG, ABA2, SOT16, and COR15B) against primary and secondary metabolites (Benjamini-Hochberg FDR, q < 0.05). Full pairwise results are given in Supplementary Table S5.

Data on carotenoids, chlorophylls, sugars, free amino acids, organic acids, phenolic compounds and GLS were taken from the parallel screening [27] and were published earlier [31]. Absolute quantitative data from the field experiment in 2021 was provided by the authors.

GLS data from the experiment investigating the effect of different irrigation regimes on pak choi were analyzed using MassHunter version 10.2 (Agilent Technologies Deutschland GmbH, Waldbronn, Germany). Subsequently, differences among the treatments were assessed by one-way ANOVA, followed by Tukey’s post-hoc test, using TIBCO Statistica 13 (StatSoft Europe GmbH, Hamburg, Germany). A significance level of *p* ≤ 0.05 was considered statistically significant.

## Results

### Environmental variation across field stations

In general, temperature as well as precipitation during the growing season decreased with increasing latitude from the subtropical field site at 44° N to subarctic field site at 68° N (Fig. 1). Conversely, photoperiod length was highest in the subarctic field site with 22–24 h daylight during the growing season, whereas in the temperate field site 18 h were recorded and 15 h in the subtropical field site. Growing season length reflected the location-specific patterns. Maturity of plants was reached after 8, 12 or 14 weeks in the subtropical, temperate or subarctic field site, respectively.

Herbivore preference was scored from 0 (no leaf damage or up to 5%) to 4 (76% to 100% leaf damage) for caterpillars of two Lepidoptera species, the specialist *Plutella xylostella* and the generalist *Mamestra brassicae*, when feeding on accessions of *B. rapa*. Leaf damage by *P. xylostella* was highest at the temperate field site (average score 2.6), followed by subtropical field site (average score 2.4) and subarctic field site (average score 1.7). Leaf damage by *M. brassicae* was also highest at the temperate field site (average score 2.24), followed by the subtropical field site (average score 1.1), while no damage was recorded at the subarctic field site (Supplemental Table 2). Related to the crop type, preference of *P. xylostella* was highest for pakchoi (average score 2.8), followed by turnips (average score 2.5) and Chinese cabbage accessions (average score 2), while preference of *M. brassicae* was highest for turnips (average score 2.4), followed by Chinese cabbage (average score 1.6) and pakchoi accessions (average score 1.3). However, due to the unbalanced number accessions for each crop type, these observations are potentially limited and represent tendencies.

### Site-associated conditions induce shifts in *B. rapa* leaf proteome

The twelve accessions were selected based on their wide geographical origin and the coverage of three crop types. Leaf proteome analysis of *B. rapa* accessions led to the identification and quantification of 5,640 proteins which were subjected to further analysis. Assessment of proteome missingness showed that over 1,700 proteins exhibited near-zero missingness across samples, while other missing values were distributed sparsely (Supplemental Fig. 1A). Comparison of raw signal distributions with left-censored median-imputed data confirmed consistent intensity profiles shifted appropriately toward lower detection thresholds without introducing quantitative distortion (Supplemental Fig. 1B).

**Fig. 2:**
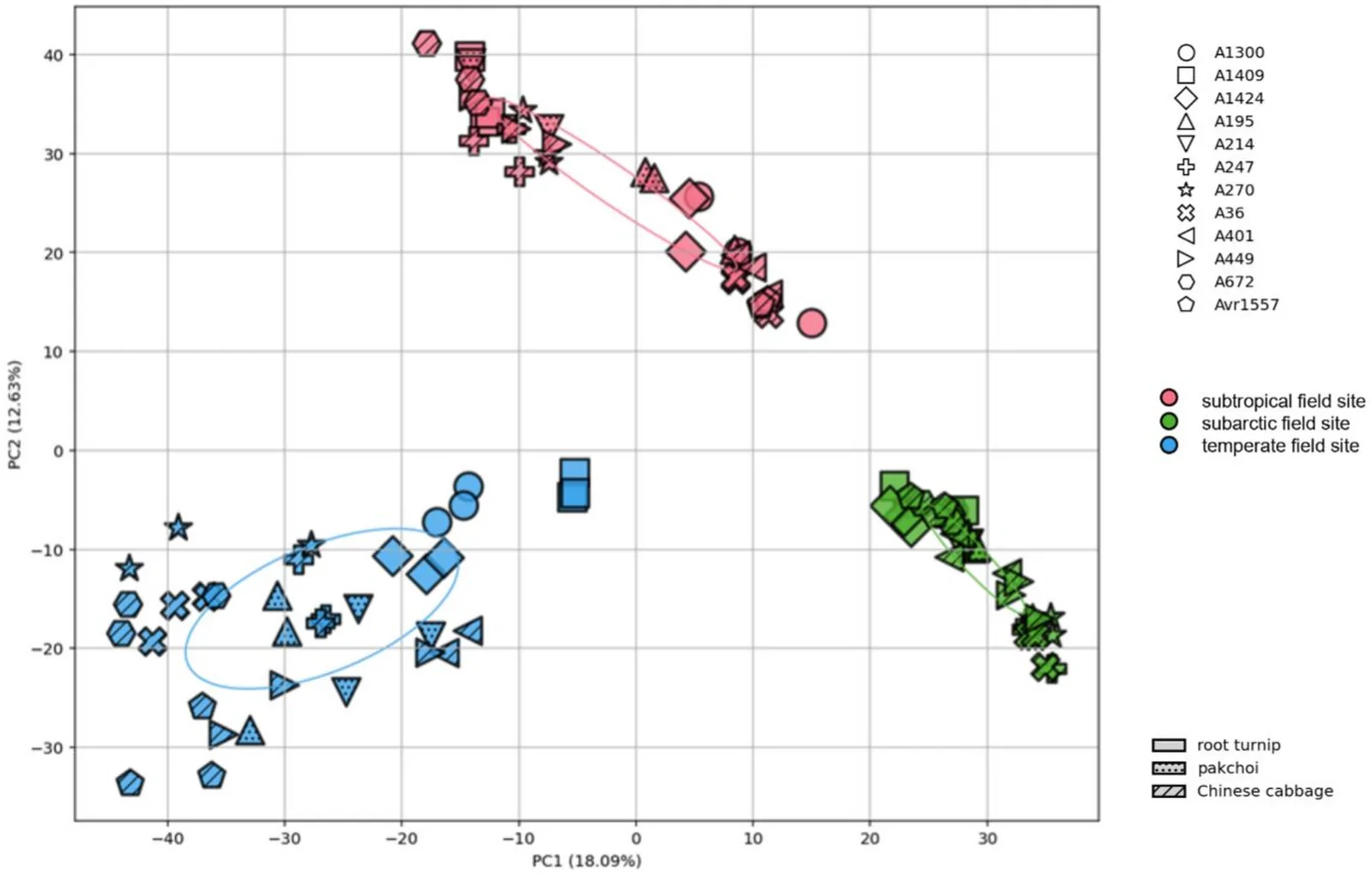
Assessment of variation in leaf proteome profiles of *B. rapa* accessions grown at subtropical, temperate or subarctic field site by principal component analysis. A distinct separation based on respective climatic conditions was found for proteome profiles. Each accession is represented by three samples, with each sample consisting of three pooled plants, per accession and field station

Principal component analysis (PCA) of *B. rapa* proteomes revealed a strong influence of geographic field sites, the first two principal components together explaining 30% of the observed variation, respectively (Fig. 2). A larger variation was found for accessions growing at the temperate field site as compared with the two other locations, indicating a more diverse proteome phenotype under these site-associated climatic condition. Here, proteome of root turnips revealed a grouping apart from the remaining crop types. Applying PLS-DA resulted in a group separation with both first components explaining 22% of the variation, (Supplemental Fig. 3). Model performance was validated by 5-fold cross-validation and a 40-iteration permutation test, resulting an observed Q2 of 0.873 and a permutation p-value of 0.024, confirming that site separation was not driven by overfitting (Supplementary Figure S18).

Joint PCA co-projection of proteome data and quantitative metabolite abundances, obtained from a parallel field experiment [31] (carotenoids, chlorophylls, sugars, free amino acids, organic acids, phenolic compounds, GLS) and GLS hydrolysis products, confirmed coordinated separation according to the field site-specific climatic condition (24.3% PC1, 19.5% PC2; Supplemental Fig. 4). Relevance networks indicated subtropical dominance in redox/GLS processes, temperate mainly on photosynthesis, and subarctic enrichment in cold-response modules (Supplemental Figs. 5–7). Direct correlations (Supplemental Table 5) linked COR15B strongly to 2-phenylethyl GLS (r = 0.718) and it’s respective hydrolysis products 3-phenylpropionitril (phenylethyl-CN; ​*r* = 0.854) and phenylethyl isothiocyanate (phenylethyl-ITC; ​*r* = 0.677), among others.

**Fig. 3:**
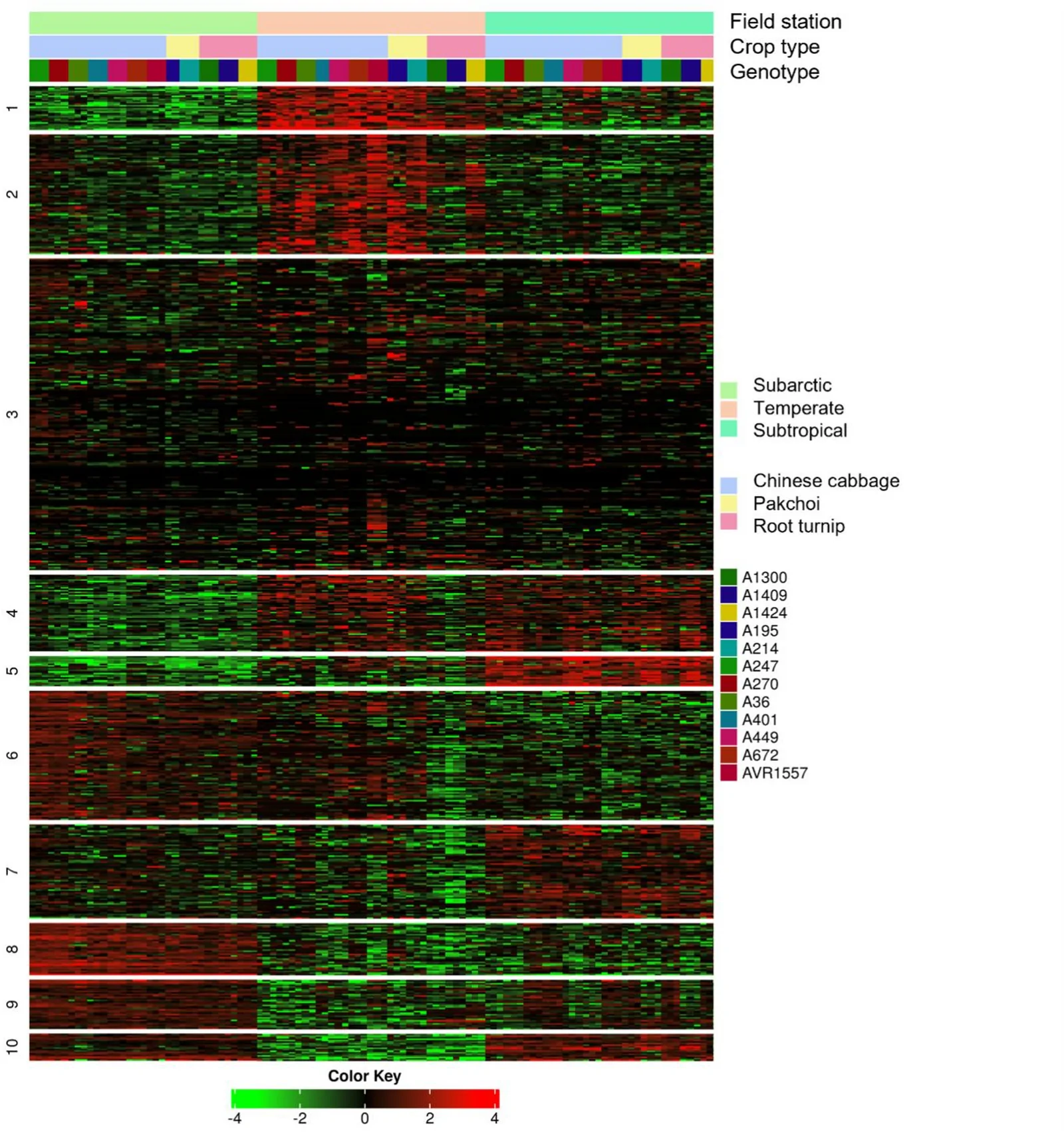
Supervised hierarchical clustering analysis of protein expression patterns. Columns represent samples of *B. rapa* accession grown at three field sites. Rows show means of normalized protein abundances from three plants per accession and field station using color coding based on a relative scale (− 4.0 to + 4.0). K-means clustering analysis identified ten clusters with specific abundance pattern

K-means clustering was applied to identify proteins that exert an abundance pattern related to climatic conditions (Fig. 3). Also, silhouette analysis indicated a global structural optimum at K = 3, the K = 10 solution was obtained for hierarchical clustering because it resolved finer-grained, biologically meaningful expression modules across field sites (Supplemental Fig. 8). To prevent circular reasoning in downstream biological interpretation, cluster assignments were validated by direct, FDR controlled differential contrasts. Hypergeometric enrichment on MapMan BINNAME categories, from climate significant proteins, confirmed strong enrichment in Photosynthesis (-log10 FDR q = 3.8), Redox homeostasis (1.83), Enzyme classification (1.60), and Protein biosynthesis (1.25).

This analysis revealed ten main clusters of protein expression, each reflecting a distinct major abundance pattern. Proteins that fall into the respective clusters, including MapMan bin codes, functional annotation and abundance values are presented in Supplemental Table 3. The abundance of three examples for field site-specific protein abundance are shown in Supplemental Fig. 9. Xanthoxin dehydrogenase (XP_033128685), known to be involved in heat stress response and abscisic acid (ABA) biosynthesis [32], is a representative cluster 5 protein expressed highest in plants grown at the subtropical field site, thiosulfate/3-mercaptopyruvate sulfurtransferase (XP_009149076), involved in the biosynthesis of sulfur-containing compounds [33], was assigned to cluster 1 and was mainly expressed in plants grown at the temperate field site. Protein COLD-REGULATED 15B (XP_009133541) confers freezing tolerance to plants [34] and was grouped to cluster 8 and expressed mainly in plants grown at the subarctic field site.

Pairwise correlations demonstrated that key protein shifts track downstream metabolic outputs (Supplementary Table S5). Subtropical-enriched ABA2 correlated positively with amino acids (valine, *r* = 0.807; threonine, *r* = 0.782) and succinic acid (*r* = 0.748). Temperate-enriched SOT16 associated with GLS hydrolysis products, including goitrin (*r* = 0.546). Subarctic-enriched COR15B strongly correlated to 2-phenylethyl GLS (*r* = 0.718) and its nitrile/isothiocyanate derivatives (*r* = 0.677 to 0.854, Supplementary Table S5), showing functional downstream metabolic alignment.

**Fig. 4:**
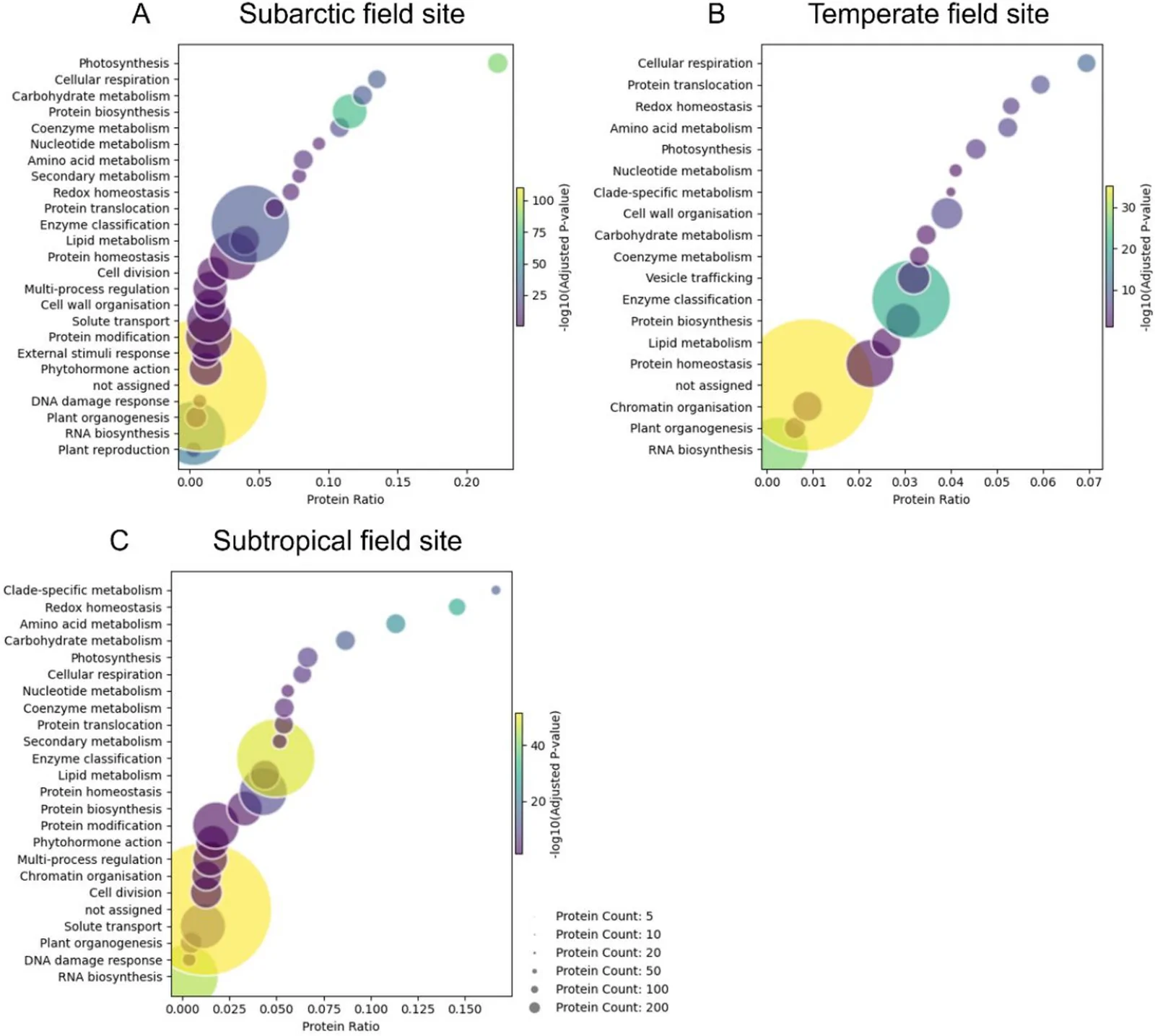
Functional enrichment analysis of all detected proteins for higher abundance in plants grown at the subarctic (**A**), temperate (**B**) or subtropical field site (**C**), based on MapMan functional assignments. The MapMan categories with the largest protein ratios are plotted in order of protein ratio. The size of the dots represents the number of proteins differentially abundant in the respective k-means cluster associated with the MapMan category. The color of the dots represents the P-adjusted values on a logarithmic scale

The proteome data set was analyzed for functional enrichment to gain insights into adaptive strategies of plants across diverse environments. For this, subarctic field site was defined as encompassing proteins in k-means clusters 6, 8 and 9, while temperate field site was defined spanning proteins in clusters 1 and 2, and subtropical field site included proteins in clusters 4, 5, 7 and 10. The top biological processes with upregulated protein enrichment scores were specific for each field site (Fig. 4). Photosynthesis, cellular respiration, carbohydrate metabolism and protein biosynthesis are prominently enriched in plants grown at the subarctic field site, likely reflecting the increased importance of energy capture under lower temperatures and longer photoperiods as well as the need for protein turnover for sustained growth under challenging climatic conditions. Compared to the subarctic site, the spread of protein ratios was narrower for plants grown at the temperate field site, suggesting more focused enrichment. A high abundance in categories such as cellular respiration, protein translocation, and redox homeostasis was found for plants grown under temperate conditions, suggesting a balance between energy production and stress management. At subtropical field site, a noticeable enrichment in clade-specific metabolism, redox homeostasis, and amino acid metabolism was observed. These alterations likely enhance defense, regulatory control and adaptation to increased biotic and abiotic challenges. The shift towards clade-specific metabolism, which constitutes mainly of GLS metabolism-related proteins, reflects an enhanced defense state of the plant and the adaptation to greater biotic and abiotic stress, such as heat and pests.

### Adaptation of cellular respiration-related proteins to environmental conditions

Cellular respiration in plant metabolism converts the chemical energy stored in organic compounds, primarily glucose, into usable energy in form of adenosine triphosphate (ATP). The main steps therein are glycolysis, tricarboxylic acid (TCA) cycle and oxidative phosphorylation. During glycolysis in the cytoplasm, glucose is split into two molecules of pyruvate, yielding a small amount of ATP and reduced electron carriers. Then, pyruvate is further broken down, producing electron carriers (NADH, FADH₂) and releasing CO₂ during TCA cycle in mitochondria. Finally, the electron carriers transfer electrons through a series of proteins, ultimately producing the majority of ATP and water as a byproduct during oxidative phosphorylation, also in mitochondria. Since the term ‘cellular respiration’ was among the top terms detected under all climatic conditions as significantly enriched (Fig. 4), proteins in this functional category were inspected in more detail.

**Fig. 5:**
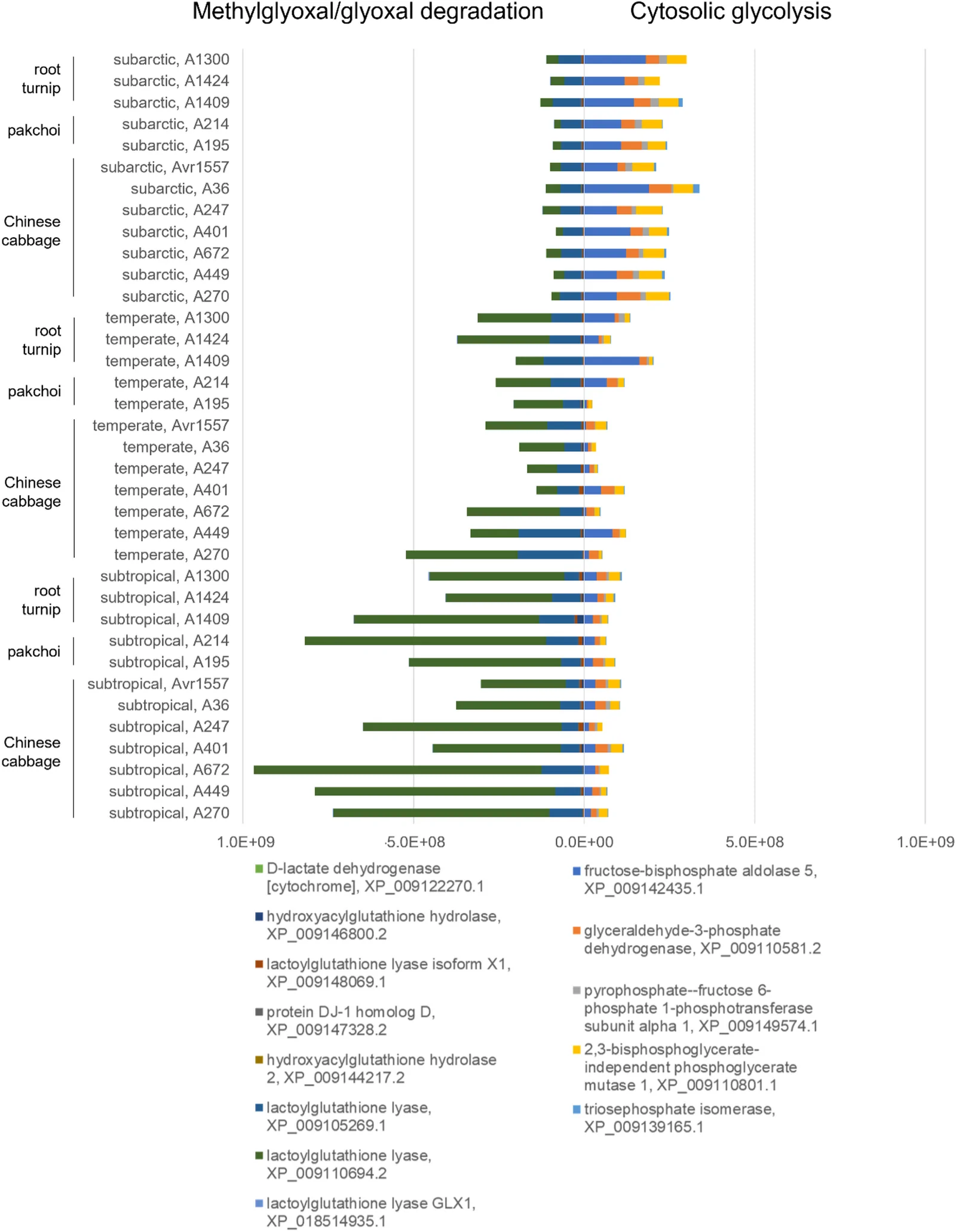
Abundance pattern of proteins related to „cellular respiration“ according to MapMan functional classification and to be involved in glycolysis. A representative selection of 5 out of 33 proteins of cytosolic glycolysis is shown. The x-axis represents protein abundance (ion intensity) as stacked bar charts and values represent means of three samples (five plants per sample) per accession and field station

A total of 198 proteins were identified in the study to be involved in cellular respiration, according to MapMan functional annotation. Forty-one proteins were related to glycolysis with most of them involved in cytosolic glycolysis and methylglyoxal/glyoxal degradation (Fig. 5). Proteins related to cytosolic glycolysis were highest abundant under subarctic condition pointing to enhanced respiration and metabolic flux to support cold acclimation under reduced enzymatic activity. In contrast, proteins involved in methylglyoxal/glyoxal degradation were upregulated under subtropical conditions, and these are responsible for removal of byproducts of glycolysis and cellular respiration. Since higher temperatures generate higher metabolic activity, the associated increase in proteins involved in glycolytic byproduct removal is essential to sustain cellular function and protect against the cytotoxic effects of these reactive metabolites in the cytoplasm.

Eleven proteins were identified that are involved in pyruvate oxidation and 41 that are involved in TCA cycle (Supplemental Fig. 10). In general, these showed a rather accession-specific abundance pattern, which was not strongly influenced by field site-associated climatic conditions.

**Fig. 6:**
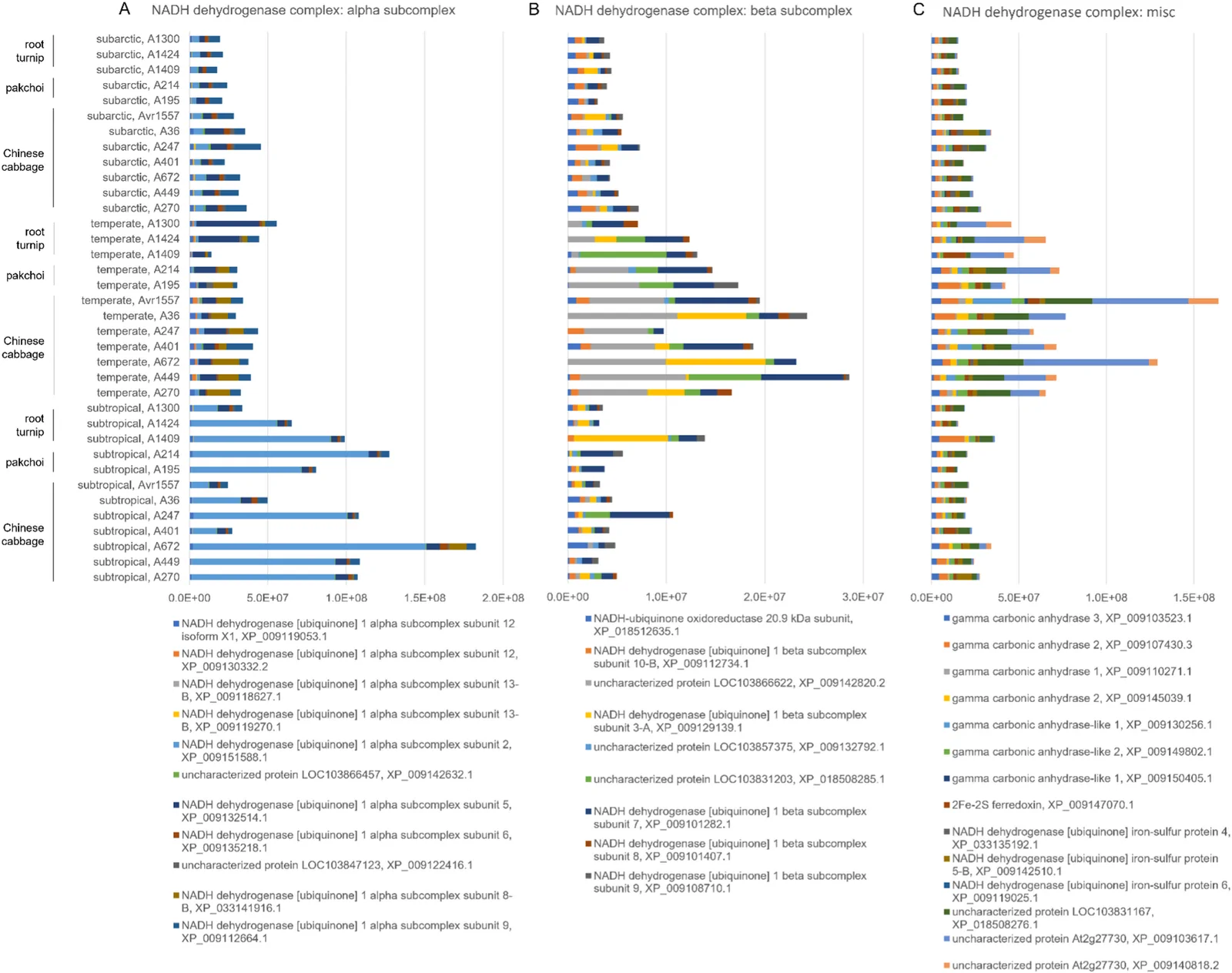
Abundance pattern of proteins related to „cellular respiration“ according to MapMan functional classification and related to NADH dehydrogenase subcomplex alpha (**A**), beta (**B**) and miscellaneous, including carbonic anhydrase (**C**). The x-axis represents protein abundance (ion intensity) as stacked bar charts and values represent means of three samples (five plants per sample) per accession and field station

The last step of ‘cellular respiration’ is the oxidative phosphorylation in the inner mitochondrial membrane [35, 36]. Involved in this process are five multi-subunit protein complexes as well as ubiquinone and cytochrome *c* as electron carriers. Complex I is the NADH dehydrogenase complex which is structurally attached to carbonic anhydrase. Complex II is succinate dehydrogenase, which links TCA cycle to the electron transfer chain. Complexes III_2_ and IV refer to cytochrome *c* reductase and cytochrome *c* oxidase, respectively. Finally, the proton gradient established by complexes I-IV is dissipated by ATP synthase (complex V) and ATP is generated. Numerous proteins involved in NADH dehydrogenase activity revealed climate dependency and most of them were highest abundant in plants grown at the temperate field site (Fig. 6). These include NADH dehydrogenase 1 alpha subcomplex subunit 12 (XP_009130332.2, B17-2) and subunit 8-B (XP_033141916.1, PGIV), uncharacterized protein LOC103866622 (XP_009142820.2, ESSS), uncharacterized protein LOC103831203 (XP_018508285.1, SGDH), uncharacterized protein At2g27730 (XP_009103617.1, P2) and uncharacterized protein At2g27730 (XP_009140818.2, P2). Under subtropical condition, NADH dehydrogenase 1 alpha subcomplex subunit 2 (XP_009151588.1, B8) was highly abundant, indicating an increased protein turn-over since this protein is located at the matrix peripheral arm of complex I, together with NADH binding site proteins (XP_009131078.1, XP_009134423.1, XP_009139726.2) as well as ubiquinone binding sites (XP_009125829.1, XP_009104553.1), and the latter abundances were not affected by climate conditions (Supplemental Table 3). Under subarctic condition, NADH dehydrogenase iron-sulfur protein 4 (XP_033135192.1, 18 kDa) was enriched, which is also located at the peripheral arm of complex I.

Proteins involved in formation of complexes III_2_, IV and V did not exhibit an abundance pattern dependent on the field site (Supplemental Fig. 11).

### Proteins involved in phytohormone metabolism mirror field site adaptation

Phytohormones play a central role when plants acclimate to environmental conditions since they activate signaling cascades that lead to physiological adjustments. However, the majority of research in this field has been conducted either in reductionistic approaches or under short-term exposition of plants to environmental changes. In our study, the term “phytohormone action” was enriched in plants grown at each field site (Fig. 4), indicating an orchestrated expression pattern of proteins involved in phytohormone metabolism. Hence, the data set was searched and revealed the expression of proteins involved in several key phytohormones. Abscisic acid (ABA) is widely recognized as the primary hormone mediating plant responses to drought, salinity, cold, and other abiotic stresses, and the nine proteins found in the study revealed a higher abundance in plants grown at the subarctic and subtropical field site, as comparted to temperate-condition associated field site (Supplemental Fig. 12A). The ABA receptor PYR1 (XP_009136879.1) was detected only in plants grown at the subarctic field site, while a different isoform of ABA receptor PYR1 (XP_009114328.1) was detected in plants grown at all field sites, although at higher levels at subarctic site. Ethylene plays a crucial role in modulating plant responses to drought and heat, including senescence. The abundance of the respective eight proteins was considerably lower in plants grown at the subarctic field site as compared to the other ones (Supplemental Fig. 12B). Jasmonic acid (JA) is central to plant defense against abiotic stresses such as drought, salinity, and cold, as well as biotic stresses. The abundance of the twelve proteins found to be involved in JA metabolism was slightly higher under subtropical condition as compared to the other ones (Supplemental Fig. 12C). Cytokinins are involved in maintaining photosynthetic activity, delay senescence, and support growth under stress, especially in combination with ABA and ethylene. Abundance of three cytokinin-related proteins showed a clear accumulation in plants grown at subarctic field site, especially when comparted to the subtropical field site (Supplemental Fig. 12D). Gibberellins are involved in growth regulation and stress adaptation, and are known to interact with ABA and ethylene. The three proteins found in the data set were clearly more abundant in plants grown at the temperate field site, as compared to the other ones (Supplemental Fig. 12E). Brassinosteroids enhance tolerance to multiple stresses by modulating antioxidant defenses, photosynthesis, and growth, often acting synergistically with other phytohormones. Only selected accessions showed an accumulation of four related proteins when grown under subtropical condition (Supplemental Fig. 12F). Auxins (indole-3-acetic acid, IAA) regulate root architecture, growth, and development under stress, and interact with other hormones to fine-tune adaptation responses. Eleven proteins were found to be involved in auxin metabolism and response, and showed highest abundance in plants grown at subtropical field site, as compared with the other ones (Supplemental Fig. 12G).

To sum up these results, proteins involved in ethylene (temperate, subtropical), cytokinin (subarctic), gibberellin (temperate) and auxin metabolism (subtropical) were the most responsive proteins related to long-term exposition of *B. rapa* to contrasting site-associated climatic conditions.

### Glucosinolate metabolism-related proteins are differentially affected by site-associated climatic conditions

A prominent group of secondary metabolites almost exclusively found in the order Brassicales are GLS. These compounds are particularly interesting for vegetable production due to their human health promoting effects as well as their role in plant defense strategies. The enrichment analysis revealed an accumulation of GLS metabolism-related proteins under subtropical condition (Fig. 4); hence, this group was inspected in more detail. GLSs can be classified according to their respective amino acid precursors into aliphatic (methionine), indole (tryptophane) and benzenic (phenylalanine and tyrosine) GLS and successive steps in the biosynthesis include elongation of side chain, core structure formation and side chain modification with different sets of enzymes involved [37]. According to MapMan annotation, 15 proteins were found to be involved in aliphatic GLS formation and these were highest abundant in plants grown at the temperate field site. Fourteen proteins were found to be responsible for indole and benzenic GLS formation and these were highest abundant in plants grown at the subtropical field site (Supplemental Fig. 13).

As GLS profiling data was available for the same accessions from a parallel field screen [31], metabolite data was compared with the abundance of GLS biosynthetic proteins. The pattern observed for protein expression was well reflected by the distribution of GLS metabolites, with aliphatic GLS being predominantly formed when plants were cultivated at the subarctic and temperate field sites, while indole/benzenic GLS were predominantly formed under subtropical-associated climatic conditions (Supplemental Fig. 14). GLS are sulfur-containing compounds and sulfur availability greatly affects GLS levels [38]. The analysis identified eight proteins involved in the sulfation of desulfo-GSL​ during core structure formation, either via glutathione (gamma-glutamyl peptidases) or 3′-phosphoadenosine 5′-phosphosulfate (sulfotransferases), and these were highest abundant in plants grown at the subtropical field site, followed by subarctic field site, and lowest abundant under temperate-associated condition (Supplemental Fig. 15).

Biological activity of GLS is exerted by their breakdown products and a high number of proteins is involved in this complex degradation. Myrosinase, a thioglucoside glucohydrolase, initiates the breakdown of intact GLS when tissue is disrupted and compartmentation is removed. While releasing glucose, a thiohydroximate-*O*-sulfate is formed, which can spontaneously degrade to isothiocyanates or nitriles. Occurrence and activity of the epithiospecifier protein leads to the generation of epithionitriles from alkenyl-GLS aglucones as well as of nitriles from non-alkenyl-GLS aglucones. Nitrile specifier proteins redirect GLS hydrolysis to the formation of simple nitriles. A number of 31 proteins related to ​GLS breakdown were found and the abundance pattern did not show a field site-specific pattern, indicating that breakdown of GLS is rather environmentally stable in *B. rapa* (Supplemental Fig. 16).

### Irrigation does not affect GLS accumulation in pakchoi

As precipitation varied among the three field sites during the cultivation period, the potential effect of different irrigation regimes (WL, 100, 80, 60 and 40%) on GLS accumulation in pakchoi was investigated under controlled growth conditions. In total, nine GLS were detected and quantified. Five GLS were classified as aliphatic GLS (3-butenyl GLS, (*R*)-2-OH-3-butenyl GLS, 4-pentenyl GLS, (*R*)-2-OH-4-pentenyl GLS, ​5-(methylsulfinyl)pentyl GLS). Three indole GLS (4-hydroxyindol-3-ylmethyl GLS, 4-methoxyindol-3-ylmethyl GLS, 1-methoxyindol-3-ylmethyl GLS) and one benzenic GLS (2-phenylethyl GLS) were identified. Total GLS levels ranged from 7.9 ± 2.3 to 10.4 ± 0.6 µmol g^− 1^ dry matter. No significant differences were observed among the irrigation levels in either total GLS levels or the levels of the three GLS classes (Supplemental Fig. 17).

### Root turnip accessions have a lower proteome plasticity as leafy accessions

We have selected twelve *B. rapa* accession covering three crop types (Chinese cabbage, pakchoi, turnips) to identify general pattern in proteome plasticity. It was also of interest, whether specific crop types are prone to high or low levels of proteome plasticity. Therefore, proteome plasticity was calculated across the different field sites within this pool of accession. The analysis revealed that proteome plasticity ranges from 0.660 to 0.741 across genotypes and shows clear crop-type trends (Supplemental Fig​. 18). Chinese cabbage cultivars occupied the higher plasticity spectrum, led by accessions A247 (PIv = 0.741) and A270 (PIv = 0.732), alongside pakchoi A195 (PIv = 0.722). In contrast, root turnip cultivars consistently exhibited the lowest plasticity, reaching a minimum in accessions A1300 (PIv = 0.660), A1424 (0.669), and A1409 (0.676). This could indicate that lower plasticity in root turnips reflects selection for storage organ sink strength and physiological buffering, whereas leafy crop types require higher proteomic flexibility to acclimate foliage development to varying thermal and photoperiod regimes.

## Discussion

Plant plasticity allows flexible restructuring of molecular networks and cellular functions in response to environmental change. This adaptability is crucial for plant resilience under stress and for evolution in unpredictable climates [39]. In this context, proteome profiles can act as molecular fingerprints for plant adaptation, revealing which processes are prioritized under different environmental pressures and identifying sensitive physiological bottlenecks. Also, cross-validation (Q2 = 0.873, permutation *p* = 0.024) and FDR-controlled statistical contrasts confirms that the observed proteomic separation across latitudinal field sites reflects genuine biological acclimation rather than statistical artifacts or model overfitting. Despite the need for a better description of climatic variation of plant traits, latitudinal gradients are a yet underutilized tool in developing climate-resilient crops. Especially for crop species with limited resources for functional genetic analyses, such as *B. rapa*, using geographic gradients to map adaptation processes can accelerate plant breeding practices.

This study made use of three contrasting field sites representing subarctic (68°N), temperate (59°N), and subtropical (44°N) climate-associated conditions. Soil-related factors, local microclimate, plant-microbe and plant-herbivore interactions were not experimentally manipulated and cannot be fully separated from geographic and climatic effects in this field-based comparison. At present, it is not possible to detangle single environmental effects (temperature, soil physics, microbiota, light, etc.) and relate their effect to the proteome pattern without using multiple field sites per climate-associated condition. Nevertheless, multi-location testing is a valuable tool to identify plant response to changing climates and there is a high demand of field laboratories in realistic crop environments [40]. This demand has been repeatedly raised [41] and while such efforts are undertaken in cereal research for quite some time, vegetable research is lacking behind. Joint PCA and relevance networks indicates that climate adaptation drives coordinated metabolic restructuring balancing subtropical redox defence, temperate photosynthetic efficiency, and subarctic cryoprotection (Supplemental Figs. 4–7). This study demonstrated the range of proteome plasticity in *B. rapa*, in response to growth at subarctic, temperate and subtropical field sites. Previous studies on *B. rapa* ssp. *pekinensis* focused on short-term responses of the transcriptome on cold stress [42] and heat stress [43, 44], or meta-analysis [45]. No general overlap with earlier findings was identified in this study and the reason is probably the physiological difference between short-term stress treatments as reported earlier versus a global metabolic reprogramming during plant development [46].

Three main pathways (cellular respiration, phytohormone metabolism, GLS metabolism) were investigated in detail to isolate field site-specific protein pattern. Plants grown at the subarctic field site showed an increased abundance of proteins involved in glycolysis, which points to a compensation for low enzymatic activities by increasing protein content of critical enzymes for cellular respiration. It is known that starch and carbohydrate levels increase under cold stress, likely for osmoprotective function and membrane stabilization [47], thus, leading to a higher demand to process these carbohydrates via glycolysis and respiration. The mitochondria respiration chain is highly temperature sensitive and cold condition leads to low ATP production rate [48]. This study identified NADH dehydrogenase iron-sulfur protein 4 (XP_033135192.1, 18 kDa) as induced in plants grown at subarctic environmental condition. This protein is involved in the oxidative phosphorylation and was found to be critical for the adaptation to cold conditions in *​A. thaliana* [49]. Loss-of-function mutants revealed high levels of electrolyte leakage, reactive oxygen species accumulation and increased sensitivity to osmotic stress, indicating that maintaining enzymatic activity of this protein by upregulating its expression is an important adaptation to cold condition also in *B​. rapa*.

With respect to phytohormone metabolism, this study identified the reduced abundance of proteins involved in ethylene metabolism in plants grown at the arctic field site, while cytokinin metabolism-related proteins were enhanced. Crosstalk among phytohormones is important for the activation of downstream genes responsible for sustained growth under cold stress [50]. The crosstalk between ethylene and cytokinins has been demonstrated in *​A.​ thaliana*, where increased ethylene content was negatively correlated with freezing tolerance, vice versa to cytokinin content [51]. A third player in this game is ABA and two isoforms of ABA receptor PYR1 (XP_009136879.1, XP_009114328.1) were specifically abundant in plants grown at the subarctic experimental site. ABA can induce the expression of cold-regulated genes [52], while the underlying mechanism of ABA signaling in the regulation of cold tolerance remains to be investigated. Gibberellins are known to promote plant growth and highest abundance of gibberellin-regulated proteins was detected in plants grown at the temperate field site, indicating that this could be the most suitable growth environment for the selected *​B. rapa* accessions. Gibberellin metabolism is tightly regulated by environmental cues as well as by interaction with other phytohormones [53]. Ethylene, cytokinins and ABA are negative regulators for gibberellin activity, while auxin seems to be a positive regulator [53]. When considering the results of this study, it is apparent that a strong negative interaction between ABA and gibberellin signaling pathways might be functional in *B. rapa*. Auxin is another positive growth regulator affecting a multitude of plant developmental processes [54]. Biosynthetic genes are induced under heat stress [54] and this is reflected by the finding of increased abundance of auxin-regulated proteins in *B. rapa* grown at subtropical condition, leading to an accelerated growth rate. Hence, the proteome analysis of phytohormone metabolism-related and phytohormone-regulated proteins delivers a comprehensive picture of crosstalk and feed-back regulation.

The accumulation of plant secondary metabolites is strongly influenced by environmental conditions [55]. GLS are plant-protective compounds that are also valued for their disease-preventive attributes in the human diet [18]. Studies in *B. oleracea* [56] and *B. rapa* [31] have shown that cold climatic conditions reduce the levels of GLS, opposite to what has been found in *​A. thaliana* [57]. Concomitant, the lowest abundance of proteins involved in GLS biosynthesis was found in plants grown at the subarctic field site. However, not only the overall amount of GLS is affected, also the composition is changed: while indole GLS are the prevalent group formed in plants grown at the subtropical field site, plants grown at the arctic and temperate field site formed mainly aliphatic GLS [31]. This is reflected by the presented results and proteins involved in aliphatic GLS biosynthesis were highest under temperate-associated field condition, while proteins involved in indole GLS biosynthesis were highly abundant under subtropical-associated field condition. Aliphatic and indole GLS have differential effects on Lepidopteran herbivores, since feeding of generalists *Manduca sexta* and *Trichoplusia ni* is negatively affected by aliphatic GLS, while indole GLS have antifeedant effects on the generalist *Myzus persicae* [58]. Artificial infestation of *​B. rapa* by the specialist *P. xylostella* lead to an induction of aliphatic GLS [31], underlining the plant protective function of aliphatic GLS. Field scoring of herbivory preference of the specialist *P. xylostella* and the generalist *M. brassicae* reported here showed that the score was similar when plants were grown at the temperate-associated or subtropic-associated conditions for *P. xylostella*, while *M. brassicae* preferred plants grown at the temperate field site. This pattern was consistent when a total of 100 *B. rapa* accessions were investigated on the same three field sites, consisting of Chinese cabbage (40 accessions), pakchoi (21 accessions), tatsoi (7 accessions), wutacai (1 accession), mizuna (5 accessions), leafy (6 accessions) and root turnip (20 accessions) [31]. This could indicate that indole GLS have an antifeedant effect on the generalist *M. brassicae*. The accumulation of indole GLS in plants grown at the subtropical field site could also be related to the accelerated growth of plants, since indole GLS contribute via indole-3-acetaldoxime and indole-3-acetonitrile metabolic intermediates to auxin biosynthesis [59] and auxin metabolism is concerted upregulated under this climatic condition. It has been shown for *B. oleracea*, forming predominantly aliphatic GLS, that photoperiod and temperature positively affect GLS biosynthesis [60]. As differential water supply did not affect GLS accumulation, the effect of growth temperature and light regime, individually and combined, on GLS metabolism in *B. rapa* need to be researched in more detail.

## Conclusion

This study demonstrates that *B. rapa* exhibits pronounced proteome plasticity across subarctic, temperate, and subtropical environments, reflecting climate-dependent reprogramming of cellular respiration, phytohormone metabolism, and GLS biosynthesis. The results highlight that adaptation to cold goes along with an enhanced abundance of proteins involved in glycolytic activity and mitochondrial function, alongside a shift in phytohormone-related protein abundance, favoring cytokinin and ABA signaling. In contrast, temperate conditions promote gibberellin-regulated protein abundance and, thus, likely growth processes, while subtropical conditions induce auxin-related protein abundance-mediated responses and increased indole GLS accumulation. These findings emphasize that proteomic profiling provides a powerful approach to develop hypothesis for unravelling the molecular mechanisms underlying plant adaptation across geographic gradients.

## Supplementary information

Supplementary Material 1

Supplementary Material 2

Supplementary Material 3

Supplementary Material 4

Supplementary Material 5

Supplementary Material 6

## Data Availability

Data underlying this article is available in the article and in its online supplementary material. Proteome raw data has been deposited at MassIVE (https://massive.ucsd.edu/ProteoSAFe/static/massive.jsp? redirect=auth) under the dataset ID MSV000100828.

## Abbreviations

ABA: abscisic acid
ATP: adenosine triphosphate
GLS: glucosinolate
FADH_2_: dehydrogenated flavin adenine dinucleotide
IAA: indole-3-acetic acid
JA: jasmonic acid
NADH: reduced nicotinamide adenine dinucleotide
PCA: principal component analysis
PLS-DA: partial least squares-discriminant analysis
TCA: tricarboxylic acid
VIR: N.I. Vavilov All-Russian Institute of Plant Genetic Resources
