# Supplementary material for: Field site-specific proteome remodeling under contrasting environmental conditions in Brassica rapa
Source: BMC Plant Biol. 2026 Nov 11;26:1929. doi: 10.1186/s12870-026-10380-5 (PMC13753453; doi:10.1186/s12870-026-10380-5)
Supplement: Supplementary file 1 [file 12870_2026_10380_MOESM1_ESM.docx]

Supplemental Figures

Supplemental Figure 1. Proteomics missingness frequency distribution and log2 intensity density (raw versus median-imputed). Missingness was quantified per protein across the 105 proteome runs (zeros and NaN treated as missing). A) shows the frequency distribution of missingness percentages. B) contrasts the raw log2 intensity distribution with a median-imputed left-censored proxy.

A

B

Supplemental Figure 2. Factorial model differential expression and crop-type adjustment. (A) Differential expression volcano plot derived from a factorial model (Field station + Genotype + Field station x Genotype + Crop-type) using Type-III contrasts and Benjamini-Hochberg FDR correction. (B) Adjusted mean aliphatic glucosinolate concentrations across crop types, accounting for unbalanced sample sizes (seven Chinese cabbage, two pakchoi, and three root turnip accessions).

Supplemental Figure 3: Assessment of variation in proteome profiles of *B. rapa* accessions grown under subtropical, temperate or subarctic climatic condition by Partial least squares-discriminant analysis (PLS-DA). Each genotype is represented by three plants per genotype and field station.

Supplemental Figure 4. Joint multi-omics projection (PCA of top-variance proteins + metabolites). A joint principal-component analysis was performed on the highest-variance proteomic features co-projected with all targeted metabolites. The first two components explained 24.3 % and 19.5 % of the variance and separated samples according to climate.

Supplemental Figure 5. Relevance network of metabolites (green nodes) and proteins (blue nodes) in plants grown at the subarctic field site. Correlations were computed exclusively on the subarctic samples using the same thresholds as above. The network is dominated by associations between cold-responsive proteins, glycolytic enzymes and metabolites related to energy metabolism and cryoprotection.

Supplemental Figure 6. Relevance network of metabolites (green nodes) and proteins (blue nodes) in plants grown at the temperate field site. The same correlation filtering criteria as in Supplementary Figure S13 were applied to the temperate data set. The resulting network shows a distinct pattern of protein–metabolite links, with stronger involvement of photosynthetic and carbohydrate-metabolism proteins compared with the subtropical network.

Supplemental Figure 7. Relevance network of metabolites (green nodes) and proteins (blue nodes) in plants grown at the subtropical field site. Pearson correlations were calculated between all detected proteins and metabolites within the subtropical samples only; edges with absolute correlation greater than 0.5 and p less than 0.05 were retained. The network reveals numerous positive and negative associations, particularly between glucosinolate-related metabolites and proteins involved in secondary metabolism and redox processes.

Supplemental Figure 8. PLS-DA permutation test, silhouette cluster validation and MapMan BINNAME enrichment of field site-significant proteins (A). PLS-DA was validated by 5-fold cross-validation and 40 random permutations of the climate labels (observed Q squared = 0.873, permutation p = 0.024). Silhouette analysis indicated a global optimum at K = 3; however, the original K = 10 solution was retained for hierarchical clustering because it resolves the finer-grained, biologically meaningful expression modules that are discussed in the main text and shown in Figure 3 (B). Hypergeometric enrichment was performed on MapMan BINNAME categories of proteins that passed FDR-controlled climate contrasts (independent of K). The most strongly enriched categories were Photosynthesis (–log10 FDR q = 3.8), Redox homeostasis (1.83), Enzyme classification (1.60) and Protein biosynthesis (1.25) (C).

Supplemental Figure 9: Pattern of field site-affected protein abundance. Xanthoxin dehydrogenase (xp_033128685) is a representative cluster 5 protein, thiosulfate/3-mercaptopyruvate sulfurtransferase (xp_009149076) was assigned to cluster 1 and protein COLD-REGULATED 15B (xp_009133541) was grouped to cluster 8. Values represent means of three plants per genotype and field station, error bars give the standard deviation.

Supplemental Figure 10: Abundance pattern of proteins related to „cellular respiration“ according to MapMan functional classification and to be involved in pyruvate oxidation (A) and TCA cycle (B). The x-axis represents protein abundance as stacked bar charts and values represent means of three plants per genotype and field station.

Supplemental Figure 11: Abundance pattern of proteins related to „cellular respiration“ according to MapMan functional classification and to be involved in oxidative phosphorylation: Alternative NAD(P)H dehydrogenase activities (A), cytochrome c oxidase/reductase complex (B) and ATP synthase complex (C). The x-axis represents protein abundance as stacked bar charts and values represent means of three plants per genotype and field station.

Supplemental Figure 12: Abundance pattern of proteins related ABA (A), ethylene (B), JA (C), cytokinins (D), gibberellins (E), brassinosteroids (F) and auxin (G) according to MapMan functional classification. The x-axis represents protein abundance as stacked bar charts and values represent means of three plants per genotype and field station.

Supplemental Figure 13: Abundance pattern of proteins related to „clade-specific metabolism“ according to MapMan functional classification and to be involved in biosynthesis of aliphatic or indole/benzenic GLS. The x-axis represents protein abundance as stacked bar charts and values represent means of three plants per genotype and field station.

Supplemental Figure 14: Shown are sums of aliphatic GLS (3-butenyl GLS, (R) 2-OH-3-butenyl GLS, 4-pentenyl GLS, (R) 2-OH-4-pentenyl GLS, 1-methylpropyl GLS, 5-(methylsulfinyl)pentyl GLS), benzenic GLS (benzyl GLS, 2-phenylethyl GLS) and indole GLS (4-hydroxyindol-3-ylmethyl GLS, indol-3-ylmethyl GLS, 4-methoxyindol-3-ylmethyl GLS, 1-methoxyindol-3-ylmethyl GLS) given as percentage of total GLS detected. Values represent the mean of three represent means of three plants per genotype and field station, and data was published earlier from a parallel screening (Artemeva *et al*., 2023).

Supplemental Figure 15: Abundance pattern of proteins related to „clade-specific metabolism“ according to MapMan functional classification and to be involved in sulfur metabolism-related biosynthesis of GLS. The x-axis represents protein abundance as stacked bar charts and values represent means of three plants per genotype and field station.

Supplemental Figure 16: Abundance pattern of proteins related to „clade-specific metabolism“ according to MapMan functional classification and to be involved in breakdown of GLS. The x-axis represents protein abundance as stacked bar charts and values represent means of three plants per genotype and field station.

[µmol g-1 dry matter]

|  | WL | 100 % | 80 % | 60 % | 40 % |
| --- | --- | --- | --- | --- | --- |
| Aliphatic GLS | 7.22458 | 9.39738 | 8.50613 | 8.69542 | 9.20109 |
| Benzenic GLS | 0.591013 | 0.814468 | 0.496262 | 0.641936 | 0.688777 |
| Indole GLS | 0.115093 | 0.172687 | 0.171807 | 0.0599529 | 0.117891 |

Supplemental Figure 17: Glucosinolate (GLS) levels [µmol g^-1^ dry matter] grouped in Aliphatic (3-butenyl GLS, (R) 2-OH-3-butenyl GLS, 4-pentenyl GLS, (R) 2-OH-4-pentenyl GLS, and 5-(methylsulfinyl)pentyl GLS), Benzenic (2-phenylethyl GLS), and Indole GLS (4-hydroxyindol-3-ylmethyl GLS, 4-methoxyindol-3-ylmethyl GLS, 1-methoxyindol-3-ylmethyl GLS) found in pak choi (*Brassica rapa* ssp. *chinensis* cv. Black behi) after 7 days of different irrigation regimes, namely waterlogged (WL), 100, 80, 60, and 40 % of water-holding capacity of the soil. Values show means + standard deviation of two independent experimental replicates (n = 2). No significant differences were found among the irrigation regimes in the levels of the three GLS classes (ANOVA, Tukey HSD test, p ≤ 0.05).

B

A

Supplemental Figure 18: Genotype Phenotypic Plasticity Index (PIv) across climates based on MinDet-imputed proteome data (A). Bar plot showing relative PIv values for twelve *B. rapa* genotypes categorized by crop type (Chinese cabbage, pakchoi, and root turnip). Chinese cabbage accession A247 showed the highest plasticity (0.741), while root turnip accession A1300 exhibited the lowest plasticity (0.660). Reaction norms of mean protein Z-scores across latitude and field site-associated climate gradients (subarctic -> temperate -> subtropical) (B). Lines represent individual genotypes colored by accession, with shape and line styles indicating crop types (Chinese cabbage, pakchoi, and root turnip). Data points represent mean protein Z-scores for each field site using MinDet-imputed proteome datasets.
