# Supplementary material for: Field site-specific proteome remodeling under contrasting environmental conditions in Brassica rapa
Source: BMC Plant Biol. 2026 Nov 11;26:1929. doi: 10.1186/s12870-026-10380-5 (PMC13753453; doi:10.1186/s12870-026-10380-5)
Supplement: Supplementary file 2 [file 12870_2026_10380_MOESM2_ESM.docx]

Supplemental Table 1: Summary of *Brassica rapa* accessions analyzed in this study.

| **Crop** | **Variety,**  **Cultivar type** | **Name** | **Origin** | **Catalog number** | **Resistance to abiotic stresses** | **Resistance to diseases** | **Resistance to *Mamestra brassicae* and *Plutella xylostella* pests** | **Biochemical composition** |
| --- | --- | --- | --- | --- | --- | --- | --- | --- |
| Chinese cabbage | Semi-heading var. *infarcta* Li, Santo, initial material of type originated from Northern China | Harumaki Shin Santousai | Japan | A270 | Resistant to cold.  Resistant to fast bolting under long day and cold conditions | Medium resistant to diseases caused by Alternaria and Xanthomonas spp.  Resistant to Plasmodiophora | Medium resistant to Mamestra and Plutella | Valuable biochemical composition: high level of ascorbic acid, pigments, phenolic compounds |
| Chinese cabbage | Heading  var. *cephalata*  f. *cylindrica* Li, Granat, initial material of type originated from North-Eastern China | Hoyo F1 | Netherlands | A449 | Non resistant to fast bolting under long day and cold conditions | Medium resistant to diseases caused by Alternaria and Xanthomonas spp. and to Plasmodiophora | Medium resistant to Mamestra and Plutella |  |
| Chinese cabbage | Leafy type  var. *dissoluta* Li, Dunganskaya, initial material of type originated from North-Western China | IT 104056 | South Korea | A672 | Resistant to abiotic stresses: cold, heat.  Resistant to fast bolting | Medium resistant to diseases caused by Alternaria and Xanthomonas spp.  Resistant to Plasmodiophora | Medium resistant to Mamestra and Plutella |  |
| Chinese cabbage | Heading  var. *cephalata*  f. *ovata* Li,  Chee-Foo, initial material from Eastern China | Kanki F1 CR 106 | Japan | A401 | Non resistant to fast bolting under long day and cold conditions. | Medium resistant to diseases caused by Alternaria and Xanthomonas spp.  Resistant to Plasmodiophora | Medium resistant to Mamestra and Plutella |  |
| Chinese cabbage | Heading with open top var. *laxa* Li, Kasin, initial material of type originated from Korea | Khasinbechu | South Korea | A247 | Resistant to abiotic stresses: cold, heat.  Resistant to fast bolting | Medium resistant to diseases caused by Alternaria and Xanthomonas spp. and to Plasmodiophora | Medium resistant to Mamestra and Plutella | Valuable biochemical composition: high level of ascorbic acid, pigments, phenolic compounds |
| Chinese cabbage | Semi-heading var. *infarcta* Li, Santo, initial material of type originated from Northern China | Khibinskaya | Russia | A36 | Resistant to fast bolting | Non resistant to diseases caused by Alternaria and Xanthomonas spp. and to Plasmodiophora | Non resistant to Mamestra and Plutella |  |
| Chinese cabbage | Heading  var. *cephalata*  f. *ovata* Li,  Matsushima, initial material from Eastern China | Mini Raioh | Japan | Avr1557 | Resistant to abiotic stresses: cold, heat.  Resistant to fast bolting | Resistant to diseases caused by Alternaria and Xanthomonas spp.  Resistant to Plasmodiophora | Resistant to Mamestra and Plutella |  |
| pakchoi | Yutsai, type spreaded in China | Mestniy | China | A195 | Resistant to cold | Medium resistant to diseases caused by Alternaria and Xanthomonas spp. and to Plasmodiophora | Medium resistant to Mamestra and Plutella | Valuable biochemical composition: high level of ascorbic acid, pigments, phenolic compounds |
| pakchoi | Taisai, type spreaded in Southern China and Japan | Nikanme Jikijiro Taisai | Japan | A214 | Resistant to heat | Medium resistant to diseases caused by Alternaria and Xanthomonas spp. and to Plasmodiophora | Medium resistant to Mamestra and Plutella |  |
| root turnip | Mammoth, initial material of type originated from UK | Afiko | Russia | A1409 | Resistant to cold | Medium resistant to diseases caused by Alternaria and Xanthomonas spp.  Resistant to Plasmodiophora | Medium resistant to Mamestra and Plutella |  |
| root turnip | Golden Ball, initial material of type originated from Japan | Golden Ball | Finland | A1424 |  | Non resistant to diseases caused by Alternaria and Xanthomonas spp.  Resistant to Plasmodiophora | Medium resistant to Mamestra and Plutella |  |
| root turnip | Gelary, initial material of type originated from Germany - Netherlands | Siloga | Germany | A1300 |  | Resistant to diseases caused by Alternaria and Xanthomonas spp.  Resistant to Plasmodiophora | Medium resistant to Mamestra and Plutella |  |
