# Supplementary material for: Field site-specific proteome remodeling under contrasting environmental conditions in Brassica rapa
Source: BMC Plant Biol. 2026 Nov 11;26:1929. doi: 10.1186/s12870-026-10380-5 (PMC13753453; doi:10.1186/s12870-026-10380-5)
Supplement: Supplementary file 3 [file 12870_2026_10380_MOESM3_ESM.docx]

Supplemental Table 2: Summary of herbivore preference of *B. rapa* accessions analyzed in this study. The scale ranges from zero (resistant) to four (susceptible) to damage by diamondback moth (*Plutella xylostella*) and cabbage moth (*Mamestra brassicae*).

| **Accession number** | **Crop** | **Name** | **Field site** | **Diamondback moth score** | **Cabbage moth score** |
| --- | --- | --- | --- | --- | --- |
| A36 | Chinese cabbage | Khibinskaya | Murmansk | 1.75 | 0 |
| A36 | Chinese cabbage | Khibinskaya | Pushkin | 4 | 2.25 |
| A36 | Chinese cabbage | Khibinskaya | Maykop | 2.3 | 1.2 |
| A195 | pakchoi | Mestniy | Pushkin | 2 | 2 |
| A195 | pakchoi | Mestniy | Maykop | 2 | 0.5 |
| A214 | pakchoi | Nikanme Jikijiro Taisai | Murmansk | 3 | 0 |
| A214 | pakchoi | Nikanme Jikijiro Taisai | Pushkin | 3 | 1.63 |
| A214 | pakchoi | Nikanme Jikijiro Taisai | Maykop | 2.9 | 0.4 |
| A247 | Chinese cabbage | Khasinbechu | Murmansk | 1.25 | 0 |
| A247 | Chinese cabbage | Khasinbechu | Pushkin | 2 | 1.5 |
| A247 | Chinese cabbage | Khasinbechu | Maykop | 2.6 | 2 |
| A270 | Chinese cabbage | Harumaki Shin Santousai | Murmansk | 1 | 0 |
| A270 | Chinese cabbage | Harumaki Shin Santousai | Pushkin | 2 | 2.63 |
| A270 | Chinese cabbage | Harumaki Shin Santousai | Maykop | 2.5 | 1.3 |
| A401 | Chinese cabbage | Kanki F1 CR 106 | Murmansk | 2 | 0 |
| A401 | Chinese cabbage | Kanki F1 CR 106 | Pushkin | 2 | 1.75 |
| A401 | Chinese cabbage | Kanki F1 CR 106 | Maykop | 3 | 2 |
| A449 | Chinese cabbage | Hoyo F1 | Murmansk | 1 | 0 |
| A449 | Chinese cabbage | Hoyo F1 | Pushkin | 3 | 2.38 |
| A449 | Chinese cabbage | Hoyo F1 | Maykop | 1.5 | 0 |
| A672 | Chinese cabbage | IT 104056 | Murmansk | 2 | 0 |
| A672 | Chinese cabbage | IT 104056 | Pushkin | 2 | 2.75 |
| A672 | Chinese cabbage | IT 104056 | Maykop | 2.3 | 0.5 |
| A1300 | root turnip | Siloga | Murmansk | 2.75 | 0 |
| A1300 | root turnip | Siloga | Pushkin | 2 | 2.25 |
| A1300 | root turnip | Siloga | Maykop | 2.7 | 2 |
| A1409 | root turnip | Afiko | Murmansk | 1.5 | 0 |
| A1409 | root turnip | Afiko | Pushkin | 3 | 2.88 |
| A1409 | root turnip | Afiko | Maykop | 3 | 2 |
| A1424 | root turnip | Golden Ball | Murmansk | 1.33 | 0 |
| A1424 | root turnip | Golden Ball | Pushkin | 2 | 2.13 |
| A1424 | root turnip | Golden Ball | Maykop | 2.1 | 1.6 |
| Avr 1557 | Chinese cabbage | Mini Raioh | Murmansk | 1 | 0 |
| Avr 1557 | Chinese cabbage | Mini Raioh | Pushkin | 2 | 1.63 |
| Avr 1557 | Chinese cabbage | Mini Raioh | Maykop | 1.5 | 0 |
