# Supplementary material for: Field site-specific proteome remodeling under contrasting environmental conditions in Brassica rapa
Source: BMC Plant Biol. 2026 Nov 11;26:1929. doi: 10.1186/s12870-026-10380-5 (PMC13753453; doi:10.1186/s12870-026-10380-5)
Supplement: Supplementary file 4 [file 12870_2026_10380_MOESM5_ESM.docx]

Supplemental Table 4: Top protein–metabolite correlations (|r| greater than 0.6, FDR less than 0.05). Pearson r, Spearman rho and Benjamini–Hochberg q-values are reported for the strongest significant pairs.

| Brassica rapa Multi‑Omics Revision Suite | | | | | |
| --- | --- | --- | --- | --- | --- |
| **Protein ID** | **Protein Name** | **Metabolite** | **Pearson r** | **Spearman ρ** | **FDR q-value** |
| XP_009105005.1 | XP_009105005.1 | GLU | 0.998 | 0.9 | 0.0000947 |
| XP_009130857.1 | XP_009130857.1 | caffeic acid | 0.998 | 0.829 | 0.000006075 |
| XP_009130857.1 | XP_009130857.1 | 3-Butenyl-ITC | 0.998 | 0.655 | 0.000008804 |
| XP_009130857.1 | XP_009130857.1 | CETPent | 0.998 | 0.655 | 0.000008804 |
| XP_009120410.1 | XP_009120410.1 | 3-Butenyl-CN | 0.997 | 0.707 | 0.0001559 |
| XP_009129509.1 | APSE | alanine | 0.996 | 0.975 | 0.0002681 |
| XP_033146241.1 | XP_033146241.1 | Methylarbutin | 0.995 | 0.771 | 0.00004543 |
| XP_009120410.1 | XP_009120410.1 | CETB | 0.995 | 0.359 | 0.0004936 |
| XP_009149240.1 | XP_009149240.1 | ASP | 0.994 | 0.9 | 0.0005141 |
| XP_009126448.1 | XP_009126448.1 | 1-Methoxyindol-3-ACN | 0.994 | 0.929 | 0.000004855 |
| XP_009130411.1 | XP_009130411.1 | valine | 0.993 | 0.6 | 0.0006634 |
| XP_009126448.1 | XP_009126448.1 | alanine | 0.993 | 0.729 | 0.000007535 |
| XP_009120063.1 | XP_009120063.1 | (Z)-Sinapic acid | 0.993 | 1 | 0.0007254 |
| XP_009120410.1 | XP_009120410.1 | 4-Pentenyl-ITC | 0.993 | 0.224 | 0.0007657 |
| XP_009110600.1 | XP_009110600.1 | alanine | 0.992 | 0.729 | 0.00001268 |
| XP_009126448.1 | XP_009126448.1 | LEU | 0.991 | 0.134 | 0.00001506 |
| XP_009126705.1 | XP_009126705.1 | Indol-3-ylmethyl GLS | -0.992 | -0.964 | 0.00001074 |
| XP_009125983.1 | UAP1/2 | β-carotene | -0.993 | -1 | 0.0006825 |
| XP_033136939.1 | DjB1/2/3 | carotenoids | -0.994 | -0.771 | 0.00005766 |
| XP_009125983.1 | UAP1/2 | Chlorophyll b | -0.999 | -1 | 0.00005605 |
