# Supplementary material for: Field site-specific proteome remodeling under contrasting environmental conditions in Brassica rapa
Source: BMC Plant Biol. 2026 Nov 11;26:1929. doi: 10.1186/s12870-026-10380-5 (PMC13753453; doi:10.1186/s12870-026-10380-5)
Supplement: Supplementary file 5 [file 12870_2026_10380_MOESM6_ESM.docx]

Supplemental Table 5: Pairwise Pearson and Spearman correlation analysis between representative protein abundances (TGG1, COR15B, SOT16, ABA2) and matched metabolite profiles, including raw p-values and Benjamini-Hochberg adjusted q-values. Pearson r, R squared, raw p-value and Benjamini–Hochberg q-value are listed for every enzyme–metabolite pair examined.

| **Target Enzyme / Protein** | **Target Metabolite** | **Pearson r** | **R2 Fit** | **Raw p-value** | **BH q-value** | **Signif.** |
| --- | --- | --- | --- | --- | --- | --- |
| XP_009133541.1 (COR15B) | phenylethyl-CN | 0.854 | 0.728 | 7.25E-11 | 5.53E-07 | *** |
| XP_009133541.1 (COR15B) | 4-Pentenyl-ITC | 0.843 | 0.711 | 2.06E-10 | 1.31E-06 | *** |
| XP_009133541.1 (COR15B) | erythronic acid | 0.791 | 0.625 | 1.60E-08 | 2.89E-05 | *** |
| XP_009133541.1 (COR15B) | 2-phenylethyl GLS | 0.718 | 0.515 | 1.23E-06 | 0.000658 | *** |
| XP_009133541.1 (COR15B) | phenylethyl-ITC | 0.677 | 0.458 | 8.04E-06 | 0.002282 | *** |
| XP_009149076.1 (SOT16) | goitrin | 0.546 | 0.298 | 0.001011 | 0.05396 | * |
| XP_009149076.1 (SOT16) | pyruvic acid | -0.416 | 0.173 | 0.01601 | 0.2341 | * |
| XP_009149076.1 (SOT16) | 2-OH-3-But-CN | 0.415 | 0.172 | 0.01626 | 0.2358 | * |
| XP_009149076.1 (SOT16) | aspartat | -0.407 | 0.166 | 0.01875 | 0.2513 | * |
| XP_009149076.1 (SOT16) | indol-3-ACN | -0.398 | 0.159 | 0.02171 | 0.2688 | * |
| XP_033128685.1 (ABA2) | valine | 0.807 | 0.651 | 1.45E-08 | 2.73E-05 | *** |
| XP_033128685.1 (ABA2) | threonine | 0.782 | 0.612 | 7.56E-08 | 8.97E-05 | *** |
| XP_033128685.1 (ABA2) | b-Phenyl-a-alanine | 0.78 | 0.608 | 8.83E-08 | 9.91E-05 | *** |
| XP_033128685.1 (ABA2) | leucine | 0.76 | 0.577 | 2.93E-07 | 0.00024 | *** |
| XP_033128685.1 (ABA2) | succinic acid | 0.748 | 0.559 | 5.72E-07 | 0.000386 | *** |
| XP_009108971.2 (TGG) | CHETPent B | 0.307 | 0.094 | 0.07324 | 0.389 | ns |
| XP_009108971.2 (TGG) | CHETPent A | 0.239 | 0.057 | 0.167 | 0.498 | ns |
| XP_009108971.2 (TGG) | quinic acid | 0.23 | 0.053 | 0.1845 | 0.5037 | ns |
| XP_009108971.2 (TGG) | 5-(methylsulfinyl)pentyl GLS | -0.228 | 0.052 | 0.1872 | 0.5091 | ns |
| XP_009108971.2 (TGG) | benzoic acid | -0.288 | 0.083 | 0.09306 | 0.4247 | ns |
| XP_009110511.1 (TGG) | 1-methoxyindol-3-ylmethyl GLS | 0.567 | 0.322 | 0.002501 | 0.1001 | * |
| XP_009110511.1 (TGG) | Phenylethyl-CN | -0.494 | 0.244 | 0.01035 | 0.1956 | * |
| XP_009110511.1 (TGG) | glyceric acid | -0.521 | 0.272 | 0.006298 | 0.1431 | * |
| XP_009110511.1 (TGG) | erythronic acid | -0.549 | 0.302 | 0.003663 | 0.1186 | * |
| XP_009122411.1 (TGG1) | asparagin | 0.317 | 0.101 | 0.1503 | 0.5431 | ns |
| XP_009122411.1 (TGG1) | succinic acid | -0.267 | 0.071 | 0.2296 | 0.6142 | ns |
| XP_009122411.1 (TGG1) | 4-methoxyindol-3-ACN | -0.287 | 0.083 | 0.1944 | 0.5859 | ns |
| XP_009122411.1 (TGG1) | a-tocopherol | -0.289 | 0.084 | 0.192 | 0.5838 | ns |
| XP_009122411.1 (TGG1) | alanine | -0.299 | 0.09 | 0.1761 | 0.5692 | ns |
| XP_009144939.2 (TGG) | goitrin | 0.427 | 0.182 | 0.01486 | 0.2079 | * |
| XP_009144939.2 (TGG) | 2-OH-3-But-CN | 0.411 | 0.169 | 0.01958 | 0.2421 | * |
| XP_009144939.2 (TGG) | methylarbutin | 0.371 | 0.138 | 0.03646 | 0.3101 | * |
| XP_009144939.2 (TGG) | asparatat | -0.319 | 0.102 | 0.07489 | 0.3898 | ns |
| XP_009144939.2 (TGG) | gamma-aminobutyric acid | -0.327 | 0.107 | 0.06766 | 0.3849 | ns |
| XP_009148652.1 (TGG) | alanine | 0.657 | 0.432 | 0.0203 | 0.2465 | * |
| XP_009148652.1 (TGG) | glyceric acid | -0.599 | 0.359 | 0.03956 | 0.3207 | * |
| XP_009148652.1 (TGG) | 4-methoxyindol-3-ylmethyl GLS | -0.601 | 0.361 | 0.03871 | 0.3207 | * |
| XP_009148652.1 (TGG) | erythrose | -0.605 | 0.367 | 0.03694 | 0.3101 | * |
| XP_009148652.1 (TGG) | glutamat | -0.617 | 0.381 | 0.03261 | 0.298 | * |
| XP_033133704.1 (TGG) | benzyl-ITC | 0.483 | 0.233 | 0.00331 | 0.1175 | * |
| XP_033133704.1 (TGG) | sec-Butyl-ITC | 0.47 | 0.221 | 0.004404 | 0.1248 | * |
| XP_033133704.1 (TGG) | 4-hydroxyindol-3-ylmethyl GLS | 0.411 | 0.169 | 0.01431 | 0.2079 | * |
| XP_033133704.1 (TGG) | alanine | 0.363 | 0.132 | 0.03198 | 0.298 | * |
| XP_033133704.1 (TGG) | erythronic acid | -0.373 | 0.139 | 0.02733 | 0.2758 | * |
| XP_033134125.1 (TGG) | asparagin | 0.463 | 0.215 | 0.005047 | 0.1373 | * |
| XP_033134125.1 (TGG) | violaxanthin | 0.433 | 0.187 | 0.009431 | 0.1832 | * |
| XP_033134125.1 (TGG) | benzyl GLS | 0.426 | 0.182 | 0.01071 | 0.1969 | * |
| XP_033134125.1 (TGG) | alanine | 0.41 | 0.168 | 0.01445 | 0.2079 | * |
| XP_033137260.1 (TGG) | phenylethyl-ITC | 0.596 | 0.356 | 0.000197 | 0.01625 | *** |
| XP_033137260.1 (TGG) | 4-pentenyl-ITC | 0.59 | 0.348 | 0.000239 | 0.01625 | *** |
| XP_033137260.1 (TGG) | erythronic acid | 0.585 | 0.342 | 0.000281 | 0.01735 | *** |
| XP_033137260.1 (TGG) | 2-phenylethyl GLS | 0.565 | 0.319 | 0.0005 | 0.02832 | *** |
| XP_033137260.1 (TGG) | benzenic GLS | 0.517 | 0.267 | 0.001755 | 0.08523 | * |
| XP_033140392.1 (TGG) | glycine | 0.626 | 0.392 | 0.000125 | 0.01625 | *** |
| XP_033140392.1 (TGG) | lutein | 0.622 | 0.387 | 0.000146 | 0.01625 | *** |
| XP_033140392.1 (TGG) | 1-methoxyindol-3-ACN | 0.608 | 0.37 | 0.000223 | 0.01625 | *** |
| XP_033140392.1 (TGG) | leucine | 0.495 | 0.245 | 0.003951 | 0.1221 | * |
| XP_033142048.1 (TGG) | threonine | 0.712 | 0.508 | 4.77E-06 | 0.002424 | *** |
| XP_033142048.1 (TGG) | serine | 0.703 | 0.495 | 7.13E-06 | 0.002424 | *** |
| XP_033142048.1 (TGG) | leucine | 0.634 | 0.403 | 9.64E-05 | 0.01625 | *** |
| XP_033142048.1 (TGG) | 4-hydroxyindol-3-ylmethyl GLS | 0.619 | 0.383 | 0.00016 | 0.01625 | *** |
| XP_033142048.1 (TGG) | alanine | 0.613 | 0.375 | 0.000193 | 0.01625 | *** |
